# Is Acute Rheumatism an Inflammatory Disease?

**Published:** 1889-07

**Authors:** T. E. Craig

**Affiliations:** Lawrenceburg, Ind.


### IS ACUTE RHEUMATISM AN INFLAMMTORY DISEASE?

BY T. E. CRAIG, M. D., LAWRENCEBURG, IND.

*Cincin. Lan.-Clin.*, March:—The author advances some very novel ideas in the course of an extremely well-written article. We abstract the following:

My views on the subject are as follows: Acute rheumatism is fever with a local manifestation in the lymph sacs of the body, just as pneumonia is a fever with a local manifestation in the cells of the lungs. It is a typical germ disease, and these germs entering the circulation through the lymphatics or lymph channels by elective affinity, find lodgement in the lymph sacs and in the lymph sacs alone. The synovial sacs are typical lymph spores. Here the germs, by their presence, excite engorgement in the capillaries of the lymph tissues, and by their vital activity give rise to ptomaines, that may set up a true inflammation in distant parts of the body, notably in the heart and pericardium, which is also a lymph sac.

I wish you to note, particularly, the difference between the joint lesions and the heart lesions in acute rheumatism. In the joints we have a simple serious effusion, containing a few epithelial cells, except when thrombi in the surrounding small blood-vessels may set up destructive changes; but in the heart and pericardium we have a true inflammation, with new tissue, adhesions and organized lymph on the valves of the heart and subcardium, as well as in a certain per cent. of cases in the pericardium.

And now I wish to call your attention to an important fact, or rather put it in the form of a question, why is it that the heart lesions are almost universally on the left side—that is, the left auricle and ventricle, with the semilunar and mitral valves—while the right side escapes? This is a rule, with few exceptions, and must have a fixed cause. My theory is that the heart lesion is caused by a poisonous principle in the blood, a ptcmaine, if you wish, and this ptomaine has been generated by the vital activity or perhaps by the death of the germs in the lymph sacs of the joints. But the ptomaine is incapable of exciting inflammation until it has been exposed to the air in the lungs. It is carried to the right side of the heart in the venous current, but does not effect the tricuspid valves nor the endocardium of the right ventricle. It is forced into the lungs and exposed to the air and returned to the left side of the heart, where it sets up a true inflammation in the structures with which it comes in contact, and sometimes, even in the heart wall itself, which is supplied with blood from the coronary arteries.

It will do no harm to refer, mentally, to our pathological anatomy and see what an inflammation is and how caused. Every inflammation arises from a lesion of the endothelium of the-blood-vessels. The lesion may be a wound, from causes without the body, or it may be caused by a morbid principle in the blood. The result is the same, whether the tissues are lacerated by a wound or a solution of continuity arises from defective nutrition or poisoned blood.

As soon as the lesion is formed, there is a rapid migration of white globules to the place. These globules break up, forming a ferment, which acting on the fibrinogen of the serum, forms fibrin, with which nature endeavors to repair the lesion. But nature does not always stop at the right point. In rheumatic carditis, we frequently have the fibrin formed in excess of the requirements of the case. The lesion may be repaired, but frequently the fibrin is piled up in the form of warty growths on the valve and endocardium and in lymph flakes and adhesive structures in the pericardium.

The point I wish to particularly impress is that the heart lesion is entirely different from the joint lesions, and hence must have a different origin.

The lymph sacs of the joints are identical in structure with the peri and endocardium, and inflammation is followed by the same results in both. But in acute rheumatism we cannot have an inflammation of the joint-structures, or we would have the same products in the synovial sacs that we have in the pericardium in pericarditis. But we never have organized lymph or fibrin in the synovial sacs—we have merely an engorgement about the joints; while we have a true inflammation of the heart structures. The engorgement arises from the presence of the germs; the inflammation from a ptomaine formed by their vital activity.—*Epitome.*

